# Factors affecting deliveries attended by skilled birth attendants in Bangladesh

**DOI:** 10.1186/s40748-017-0046-0

**Published:** 2017-03-21

**Authors:** Gulam Muhammed Al Kibria, Swagata Ghosh, Shakir Hossen, Rifath Ara Alam Barsha, Atia Sharmeen, S. M. Iftekhar Uddin

**Affiliations:** 1 Department of International Health, Johns Hopkins Bloomberg School of Public Health, 615 North Wolfe Street, Baltimore, MD 21205 USA; 2 School of Public Health, University of Saskatchewan, Saskatoon, Saskatchewan Canada; 3 Department of Pediatrics, Sylhet M. A. G. Osmani Medical College Hospital, ProjAHNMo- Johns Hopkins University-Bangladesh, Sylhet, Bangladesh; 4 Shimantik, Sylhet, Bangladesh

**Keywords:** Maternal mortality ratio, Skilled birth attendants, Factors, Delivery, Childbirth, Bangladesh

## Abstract

**Background:**

The presence of skilled birth attendants (SBAs) is crucial in childbirth to reduce the maternal mortality ratio (MMR) and to achieve the maternal mortality target of the United Nations’ Sustainable Development Goals (SDGs). The aim of this study was to investigate the factors related to childbirths attended by SBAs in Bangladesh.

**Methods:**

Data from the Bangladesh Demographic and Health Survey (2014 BDHS) were analyzed. Logistic regression was applied to calculate crude odds ratios (CORs), adjusted odds ratios (AORs), 95% confidence intervals (CIs), and p-values.

**Results:**

In Bangladesh, 35.9% of deliveries were attended by SBAs, and 44.2% of those women received at least one antenatal check-up by a skilled provider. The deliveries by SBAs were less than 50% of the total deliveries in all divisions, excluding Khulna. Known pregnancy complications (AOR: 1.2; 95% CI: 1.1–1.4), higher level of education in both women (AOR: 1.7; 95% CI: 1.2–2.3) and their husbands (AOR: 1.8; 95% CI: 1.3–2.4), receiving antenatal care (ANC) by a skilled provider during the pregnancy period (AOR: 1.5; 95% CI: 1.1–2.1), and higher wealth quintiles (AOR: 3.4; 95% CI: 2.5–4.7) were all significantly associated with an increased likelihood of a delivery by SBAs (*p* <0.05). In contrast, women living in rural areas (AOR: 0.7; 95% CI: 0.6–0.8) and the Sylhet Division (AOR: 0.4; 95% CI: 0.3–0.5) were less likely to be delivered by SBAs.

**Conclusions:**

To achieve the target of the Government of Bangladesh - 50% of deliveries to be attended by SBAs - it is important to increase ANC services and awareness programs in all seven divisions of Bangladesh. Special focus in rural areas is also required to meet this target. A new study should be conducted to explore the unexamined factors associated with the presence of SBAs during childbirth.

## Plain english summary

The maternal mortality ratio (MMR) is high in Bangladesh. The presence of skilled birth attendants (SBAs) during delivery may reduce this high MMR. To meet the MMR target of the United Nations’ Sustainable Development Goals (SDGs) it is important to increase the number of deliveries attended by SBAs. This study was conducted to examine the factors associated with deliveries attended by SBAs.

Cross-sectional secondary, and generalizable data from the Bangladesh Demographic and Health Survey (2014 BDHS) were analyzed. Factors were selected based on published reports and available data.

The factors that were found to be positively associated with deliveries attended by SBAs included known pregnancy complications higher education level of women, receiving antenatal care (ANC) by a skilled provider during the pregnancy period, and higher wealth quintile. Conversely, the factors that were inversely associated with deliveries attended by SBAs included living in rural areas and divisions other than Khulna.

A survey was recommended to investigate the associations of factors not explored in this study but that were validated in previous studies. The government should focus more on rural areas along with divisions with a low presence of SBAs during delivery. It was also recommended to focus on potential factors (e.g., ANC visit by a skilled provider and identifying pregnancy complications) to improve the number of deliveries attended by SBAs.

## Background

Despite a substantial reduction of the estimated maternal mortality ratio (MMR) in Bangladesh from 550 per 100,000 live births in 1990 to 176 per 100,000 live births in 2015 the MMR is higher than most other countries [1]. Reduction of the MMR was a target of the United Nations’ (UN) Millennium Development Goals (MDGs), and it is also stated as one of the Health Goals (Goal 3) of the UN’s Sustainable Development Goals (SDGs) SDG 3.1 aims to reduce MMR to less than 70 per 100,000 live births by 2030 [2]. The estimated annual reduction of MMR in Bangladesh from 1990 to 2015 was 5% and to achieve the targets of the SDGs this progress needs to be accelerated [3].

According to the World Health Organization (WHO) skilled birth attendants (SBAs) are accredited health professionals (such as midwives, doctors, or nurses) who have been educated and trained to proficiently manage normal (i.e., uncomplicated) pregnancies, childbirths and the immediate postnatal period, as well as handle the identification, management and referral of complications in women and newborns [3]. The presence of SBAs can significantly reduce maternal and neonatal mortality by preventing or managing most obstetric complications [4, 5] including stillbirths [6]. In addition to the high MMR, Bangladesh has a high neonatal mortality rate with an estimated 23 per thousand live-births in 2015 [7].

The latest 2014 Bangladesh Demographic and Health Survey (2014 BDHS) revealed that the percentage of deliveries attended by SBAs in Bangladesh increased from 16% in 2004 to 42% in 2014 [8]. This rate is below the targeted level (50%) of the Health Population and Nutrition Sector Development Program (HPNSDP) set by the Ministry of the Health and Family Welfare (MOHFW) of the Government of Bangladesh [9]. To increase the number of deliveries attended by SBAs in Bangladesh and to achieve the target of the HPNSDP, a clear, focused, and evidence-based approach is needed. The identification of the factors that affect deliveries attended by SBAs is important in this approach. There are few recent studies in Bangladesh that have examined the factors associated with the presence of SBAs during deliveries, which ultimately limits our understanding of this problem and indicates that this issue has been underestimated in Bangladesh. Therefore, further comprehensive studies are required.

Previous studies found that factors of different levels such as individual household or socioeconomic conditions simultaneously affect the decision of delivery attended by SBAs [10–14]. In Bangladesh, there is inequality in rural and urban areas, and inequality according to region and division. A population-based and nationally representative study may reveal the overall state of the SBA attended deliveries, which would be beneficial for the planning of health programs. It is better to address the modifiable factors and the areas or regions requiring intervention to increase the number of deliveries attended by SBAs. We attempted to identify and fill these existing gaps of knowledge to inform the policymakers and researchers to take a risk-specific intervention approach. We examined the factors that affect the decision of an SBA attended delivery by analyzing a nationally representative dataset from Bangladesh.

## Methods

### Ethics statement

We used anonymized survey data that were available for academic use ethical approval for this study was not required. We obtained approval to use the data from ICF International Rockville, Maryland, USA in August 2016.

### Data source

We analyzed secondary and cross-sectional data from the 2014 BDHS. This survey was conducted from June to November 2014 by Mitra and Associates. Details of this population-based survey including survey design methodologies, findings, and questionnaires have been described elsewhere [8].

Three types of questionnaires were used in the 2014 BDHS: a household questionnaire a women’s questionnaire, and a community questionnaire. Information from ever-married women aged 15-49 years was collected by the women’s questionnaire. A total of 164 field workers were recruited based on experience, education level, and their willingness. Then, they were trained to conduct the oral interview [8].

Women were asked questions on the following topics: background characteristics (e.g. age, education, religion, media exposure), reproductive history, use and source of family planning methods, antenatal care, delivery care, postnatal care, newborn care, breastfeeding and infant feeding practices, child immunizations and illnesses, marriage, fertility preferences, husband’s background and respondent’s work, and awareness of AIDS and other sexually transmitted infections [8].

### Sample design

To represent the demographics of women across the country the sample of the BDHS survey was nationally representative. The survey used a sampling frame from the list of enumeration areas (EAs) of the 2011 Population and Housing Census of the People’s Republic of Bangladesh, provided by the Bangladesh Bureau of Statistics (BBS) [8].

The survey was based on a two-stage stratified sample of households. In the first stage 600 EAs were selected with a probability proportional to the EA size there were 207 EAs in urban areas and 393 in rural areas. A complete household listing operation was then carried out in all of the selected EAs to provide a sampling frame for the second-stage selection of households. In this second stage of sampling a systematic sample of 30 households on average was selected per EA the sample provided statistically reliable estimates of the key demographic and health variables for the country as a whole for urban and rural areas separately, and for each of the seven divisions. With this design, the survey selected 18,000 households with the expectation of approximately 18,000 completed interviews with ever-married women [8].

The weighted distribution of urban-rural households in the survey was based on the urban-rural distribution of the 2011 population census of the country a modified urban-rural household distribution was reflected by adjusting the sample weights and any significant differences in the overall survey indicators were not expected among the population [8].

### Coverage of the sample

Initially 17,989 households were selected based on the weighting of the population distribution of rural-urban place and division. Among the selected households, 17,565 were occupied during the time of the interview, and 17,300 (99%) of the households were then interviewed. A total of 18,245 ever-married women of reproductive age (15-49years) were identified in these households, and 17,863 women were interviewed (98% response rate). Rural and urban areas had similar response rates [8].

### Participants

We examined a cohort of women among those who participated in the BDHS we minimized recall bias by including the women who had given birth to at least one child within the last five years which resulted in 6,855 deliveries within this period. For our study, we considered one delivery per woman as a single unit of analysis. In addition, as some women delivered more than once in this 5-year period, we considered their latest delivery as the delivery to be included in the analysis. In this way, 4,468 women delivered at least one child within the previous 5-year period and they were included in our analyses.

### Outcome

Women reported on their last childbirth and stated whether attendants were present during their childbirth. In our analysis we used the WHO definition to incorporate deliveries attended by SBAs.

Using this definition, SBAs included qualified doctors, nurses, midwives, family welfare visitors, and community skilled birth attendants [4]. We defined traditional birth attendants, unqualified doctors, relatives, neighbors, and others as unskilled attendants.

We then coded the birth attendant variable according to the assistance of delivery (SBA = 1 and unskilled attendant = 0).

### Exposure variables

We selected the following individual fertility and contextual factors based on published reports and data structure of the BDHS: age of the women and their husbands, parity, birth interval, previous history of deceased children, education level of the women and their husbands, occupation of the women, wealth quintile, exposure to mass media (i.e., radio, television or newspaper), receiving antenatal care (ANC) during pregnancy, division, place of residence (i.e., urban or rural), and religion.

### Statistical analyses

A contingency table was used to describe the selected background characteristics and to compare women according to the selected characteristics. Simple and multiple logistic regression analyses were applied to calculate crude (unadjusted) odds ratios (CORs) and adjusted odds ratios (AORs) respectively. The odds ratios (ORs) with 95% confidence intervals (CIs) and significance levels (*p*-value) were reported. Only variables with a predetermined significance level (*p* < 0.2) in the simple logistic regression were kept in the multiple logistic regression. Variance inflation factors (VIFs) were estimated to check collinearity. High collinearity was assumed for a VIF greater than 10.

The discrete variables (i.e. age, parity, birth interval, number of children) were converted into categorical variables. The wealth index groups were based on household assets and facilities. The ownership of assets (i.e., television, radio, fridge, car, bicycle, and motorcycle), and facilities (i.e., source of drinking water, type of toilet, electricity, and type of building materials used in the place of dwelling) were weighted using a principal component analysis (PCA). Stata 13.0 (Stata Corp, College Station, TX) was used for all data analyses.

## Results

The selected background characteristics of the participants according to birth attendant’ group (i.e. skilled or unskilled) are shown in Table 1. Among 4,468 of the women included in the study, 35.9% were attended by skilled attendants during childbirth. The majority of women were from the younger reproductive age group (54.9%), followed by the middle (39.1%) and late (6.0%) reproductive age groups. There were 73.1% of women in our study who received ANC. Regarding education, 86.5% of the women had formal education, whereas 77.1% of the husbands in the study had formal education. A vast majority of the women (78.2%) were housewives and were not involved with any formal occupation. More than two-thirds of the women were from rural areas. A similar proportion of participants were obtained from each division. Less than one-tenth of the women were heads of household. There were 92.0% of the respondents who were Muslims.

**Table 1:** Percentage distribution of women according to birth attendants and selected background characteristics

Characteristics	Unskilled BAs-N(%)^a^	SBAs-N(%)^a^	Total
Total women	2,864 (64.1)	1,604 (35.9)	4,468
Current age of women (years)			
15–24	1,585 (64.6)	870 (35.4)	2,455
25–34	1,101 (63.1)	643 (36.9)	1,744
35–49	178 (66.2)	91 (33.8)	269
Husbands’ age (years)			
15–25	446 (65.3)	237 (34.7)	683
25–40	2,014 (63.7)	1,146 (36.3)	3,160
> 40	404 (64.6)	221 (35.4)	625
Parity			
1	1,009 (55.6)	806 (44.4)	1,815
≥ 2	1,855 (69.9)	798 (30.1)	2,653
Birth Interval (years)			
0–2	247 (76.9)	74 (23.1)	321
≥ 3	2,617 (63.1)	1,530 (36.9)	4,147
Pregnancy complications			
No	1,126 (60.9)	724 (39.1)	1,850
Yes	874 (53.0)	776 (47.0)	1,650
Don't know	864 (89.3)	104 (10.7)	968
Previous history of dead children			
0	2,437 (62.5)	1,464 (37.5)	3,901
1	362 (75.3)	119 (24.7)	481
≥ 2	65 (75.6)	21 (24.4)	86
Receiving at least one antenatal care by a skilled provider			
No	1,043 (86.8)	159 (13.2)	1,202
Yes	1,821 (55.8)	1,445 (44.2)	3,266
Education level of women			
No formal education	502 (83.4)	100 (16.6)	602
Primary	946 (77.0)	282 (23.0)	1,228
Secondary	1,259 (59.5)	858 (40.5)	2,117
Higher	157 (30.1)	364 (69.9)	521
Education level of husbands of the women			
No formal education	831 (81.2)	192 (18.8)	1,023
Primary	989 (73.4)	359 (26.6)	1,348
Secondary	808 (57.4)	599 (42.6)	1,407
Higher	335 (34.2)	453 (65.8)	688
Sex of the household head			
Male	2,603 (64.1)	1,461 (35.9)	4,064
Female	261 (64.6)	143 (35.4)	404
Occupation of the women			
Not working	2,184 (62.5)	1,309 (37.5)	3,493
Working	679 (69.8)	294 (30.2)	973
Wealth quintile			
Poorest	802 (85.7)	134 (34.3)	936
Poorer	662 (77.9)	188 (22.1)	850
Middle	574 (67.0)	283 (33.0)	857
Richer	524 (55.9)	414 (44.1)	938
Richest	302 (34.1)	565 (65.9)	887
Frequency of reading newspaper			
No	2,581 (68.8)	1,171 (31.2)	3,752
Less than once a week	194 (46.7)	221 (53.3)	415
At least once a week	81 (27.9)	209 (72.1)	290
Frequency of listening to radio			
No	2,749 (64.5)	1,513 (35.5)	4,262
Less than once a week	45 (50.0)	45 (50.0)	90
At least once a week	70 (60.3)	46 (39.7)	116
Frequency of watching television			
No	1,461 (79.2)	383 (20.8)	1,844
Less than once a week	275 (70.0)	118 (30.0)	393
At least once a week	1,128 (50.6)	1,103 (49.4)	2,231
Place of residence			
Urban	692 (48.1)	747(51.9)	1,439
Rural	2,172 (71.7)	857(28.3)	3,029
Region (division)			
Khulna	250 (47.6)	275 (52.4)	525
Barisal	375 (70.5)	157 (29.5)	532
Chittagong	576 (66.9)	284 (33.1)	857
Dhaka	471 (59.7)	318 (40.3)	789
Rajshahi	334 (61.3)	211 (38.7)	545
Rangpur	353 (65.0)	190 (35.0)	543
Sylhet	508 (75.0)	169 (25.0)	677
Religion			
Islam	2,646 (64.4)	1,464 (35.6)	4,110
Others	218 (60.9)	140 (39.1)	358

^a^Row percentage

The calculated crude and adjusted ORs of the selected variables with 95% CIs and significance levels (*p*-values) are presented in Table 2. The variables with significance level up to 0.2 in the unadjusted level were included in the final adjustment to calculate the AORs. We did not find any specific variables to be highly collinear however there was a weak collinearity between ANC by a skilled health personnel and pregnancy complications, which had a VIF of 3.5.

**Table 2:** Logistic regression analyses showing the crude and adjusted odds ratios (with 95% confidence intervals) and significance level, deliveries attended by skilled birth attendants in Bangladesh

Characteristics	Crude OR (95% CI)	*p*-value	Adjusted OR(95% CI)	*p*-value
Current age of women (years)				
15–24	Ref			
25–34	1.1 (0.9–1.2)	0.3		
35–49	0.9 (0.7–1.2)	0.6		
Husbands’ age (years)				
15–25	Ref			
25–40	1.1 (0.9–1.3)	0.5		
> 40	1.0 (0.8–1.3)	0.8		
Parity				
1	Ref		Ref	
≥ 2	0.5 (0.5–0.6)	<0.001	0.7 (0.6–0.9)	<0.001
Birth interval (years)				
0–2	Ref		Ref	
≥ 3	1.9 (1.4–2.6)	<0.001	1.3 (0.9–1.8)	0.1
Pregnancy complications				
No	Ref		Ref	
Yes	1.4 (1.2–1.6)	<0.001	1.2 (1.1–1.4)	<0.05
Don't know	0.2 (0.1–0.3)	<0.001	0.6 (0.3–0.7)	<0.05
Previous history of dead children				
0	Ref		Ref	
1	0.5 (0.4–0.7)	<0.001	0.9 (0.8–1.3)	0.9
≥ 2	0.5 (0.3–0.9)	<0.05	1.5 (0.8–2.6)	0.2
Receiving at least one antenatal care by a skilled provider				
No	Ref		Ref	
Yes	5.2 (4.1–6.6)	<0.001	1.5 (1.1–2.1)	<0.05
Education level of women				
No formal education	Ref		Ref	
Primary	1.5 (1.1–2.0)	<0.001	1.0 (0.8–1.3)	0.8
Secondary	3.4 (2.6–4.5)	<0.001	1.2 (0.9–1.5)	0.3
Higher	11.6 (8.4–16.0)	<0.001	1.7 (1.2–2.3)	<0.05
Education level of husbands of the women				
No formal education	Ref		Ref	
Primary	1.6 (1.3–1.9)	<0.001	1.3 (0.9–1.4)	0.3
Secondary	3.2 (2.6–3.9)	<0.001	1.3 (1.0–1.6)	<0.05
Higher	8.3 (6.6–10.7)	<0.001	1.8 (1.3–2.4)	<0.001
Sex of the household head				
Male	Ref			
Female	1.0 (0.8–1.2)	0.8		
Occupation of the women				
Not working	Ref		Ref	
Working	0.7 (0.6–0.8)	<0.001	0.8 (0.6–0.9)	<0.001
Wealth quintile				
Poorest	Ref		Ref	
Poorer	1.7 (1.3–2.3)	<0.001	1.3 (1.0–1.7)	<0.05
Middle	2.9 (2.2–3.6)	<0.001	1.7 (1.3–2.3)	<0.001
Richer	4.7 (3.6–6.1)	<0.001	2.2 (1.6–2.8)	<0.001
Richest	11.6 (8.9–15.1)	<0.001	3.4 (2.5–4.7)	<0.001
Frequency of reading newspaper				
No	Ref		Ref	
Less than once a week	2.5 (2.0–3.0)	<0.001	1.2 (0.9–1.5)	0.1
At least once a week	5.7 (4.3–7.5)	<0.001	1.6 (1.2–2.2)	<0.05
Frequency of listening to radio				
No	Ref		Ref	
Less than once a week	1.8 (1.2–2.7)	<0.05	1.2 (0.8–1.9)	0.4
At least once a week	1.2 (0.8–1.8)	0.3	0.6 (0.4–1.0)	<0.05
Frequency of watching television				
No	Ref		Ref	
Less than once a week	1.6 (1.3–2.1)	<0.001	1.0 (0.8–1.3)	0.8
At least once a week	3.7 (3.2–4.3)	<0.001	1.2 (0.9–1.4)	0.1
Place of residence				
Urban	Ref		Ref	
Rural	0.3 (0.3–0.4)	<0.001	0.7 (0.6–0.8)	<0.001
Region (division)				
Khulna	Ref		Ref	
Barisal	0.3 (0.3–0.5)	<0.001	0.4 (0.3–0.6)	<0.001
Chittagong	0.4 (0.4–0.6)	<0.001	0.4 (0.3–0.5)	<0.001
Dhaka	0.6 (0.5–0.8)	<0.001	0.4 (0.4–0.6)	<0.001
Rajshahi	0.6 (0.4–0.7)	<0.001	0.6 (0.5–0.8)	<0.001
Rangpur	0.5 (0.4–0.6)	<0.001	0.5 (0.4–0.7)	<0.001
Sylhet	0.3 (0.2–0.3)	<0.001	0.4 (0.3–0.5)	<0.001
Religion				
Islam	Ref		Ref	
Others	1.1 (0.9–1.4)	0.2	1.1 (0.8–1.4)	0.6

The age group (of study women and their husbands) was not a significant predictor of the deliveries attended by SBAs (*p* >0.05). The women with a gap of three or more years between subsequent deliveries were 1.5 times more likely to be delivered by SBAs compared to women with a birth interval of less than two years. The higher education level of women (AOR: 1.7 95% CI: 1.2-2.3) and their husbands (AOR: 1.8 95% CI: 1.3-2.4) was a significant predictor. The women from higher wealth quintiles were more likely to be delivered by SBAs in both the unadjusted (COR: 11.6 95% CI: 8.9-15.1) and adjusted analyses (AOR: 3.4 95% CI: 2.5-4.7). Though exposure to mass media (i.e. reading newspapers, listening to radio or watching television) was a significant predictor in the unadjusted level (*p* <0.05), exposure to television did not remain significant after adjusting for other factors. The mothers who received a follow-up from an SBA during the pregnancy period increased the likelihood to be delivered by an SBA in comparison to the women who did not receive a follow up from an SBA (AOR: 1.5 95% CI: 1.1-2.1). The likelihood of a delivery attended by SBAs was significantly lower in all six divisions compared to Khulna. Religion was not significantly associated with deliveries attended by SBAs.

## Discussion

Our analyses revealed that known pregnancy complications higher education level of women, receiving ANC by a skilled provider during the pregnancy period, and higher wealth quintiles were positively associated with the deliveries attended by SBAs in Bangladesh. On the other hand, living in rural areas and divisions other than Khulna were inversely associated with the likelihood to be delivered by SBAs within the country. We did not find any association of deliveries attended by SBAs with age, history of child death, sex of the head of the household, exposure to mass media, and religion.

The education level of the husbands was positively associated with deliveries attended by SBAs in our study and this finding is supported by other studies [10–12]. This may be the result of greater knowledge and awareness of the importance of SBAs in deliveries. The association of education level may also be linked to the wealth quintile, as educated people have more opportunities to work and get well-paying jobs. Moreover, education was the strongest and most consistent predictor of health status [15]. As reported in other studies in Bangladesh, there was a significant association between women with an occupation outside of the home and deliveries by SBAs [10, 11]. However, we found the opposite there was a negative association between women working outside of the home and deliveries attended by skilled attendants. A study in Nepal found a similar association [13].

Similar to other studies we found that women from higher wealth quintiles were more likely to be delivered by skilled attendants compared to less privileged women [10, 12, 16, 17]. This may be due to the costs of SBAs during deliveries, which is difficult to manage for families with lower income. Improving the socio-economic status is difficult and a long-term process however a review of the BRAC-ICDDR,B Joint Research Project Working Paper Series in Bangladesh suggested that microcredit programs may improve maternal and child health conditions within the targeted program areas [18]. The application of improvement programs is a potential area for policymakers in the country to get involved.

We found that women living in rural areas were less likely to be delivered by SBAs. Mothers living in rural areas have less access to antenatal obstetric, and postnatal care services compared to their urban counterparts consequently women in rural areas do not utilize family planning, antenatal and other services as much as women in urban areas [8]. Less access and use of health services is closely linked to the lower socioeconomic conditions of rural people and inadequate health care services in rural areas of the country. In Bangladesh, there are vast differences between rural and urban utilization of health services there are also differences between rural and urban health-related outcomes including the neonatal and infant mortality rate and the maternal mortality ratio [8]. This inequality is required to be fulfilled by community-based programs. Moreover, more than 65% of the people of Bangladesh live in rural areas the people in rural areas should be prioritized to achieve desired health outcomes [19].

Women living in divisions other than Khulna were less likely to be delivered by SBAs. Moreover in all other divisions, less than one-third of the pregnant women were delivered by SBAs. Specifically, Sylhet had the lowest number of deliveries attended by SBAs in comparison to the other six divisions. Khulna is one of the best performing divisions for all health indicators in Bangladesh and was able to meet the governmental target for SBA attendance during childbirth compared to most of the other divisions Khulna had the highest proportion of women delivered in health facilities and the highest proportion of women receiving antenatal and postnatal care [8]. The differences in the regional use of health services contributed to the gap in health indicators and the increased number of deliveries attended by SBAs. The government should increase awareness programs in the other six divisions and intervene to increase the likelihood of being delivered by SBAs.

Receiving ANC by a skilled health care provider during pregnancy and prolonged intervals between subsequent births were positively associated with deliveries attended by SBAs and this is corroborated by the findings from other studies of Bangladesh [10–12, 14]. A prolonged birth interval is also associated with increased awareness among women. Receiving counseling and information from the SBAs during pregnancy may increase the likelihood of being delivered by SBAs [20, 21] the pregnancy period is the most suitable opportunity for intervention. Moreover antenatal counseling is one of the four pillars of the Safe Motherhood Initiative based on its effectiveness [22].

A study in Nepal found an association between the autonomy of women and deliveries attended by SBAs [23]. However we did not find any association between head of household and deliveries attended by SBAs.

We considered that women who have been exposed to mass media (i.e. radio, television, and newspaper) were more likely to use SBAs during delivery. A study in Uganda found an association between exposure to mass media and birth preparedness [24] therefore we considered that women may become more aware of SBAs by advertisements or exposure to awareness programs. Moreover, one study in Bangladesh found a positive association between the use of contraceptives and television exposure [25]. Television exposure appeared to be a significant predictor in the calculated CORs however television exposure did not remain significant when we adjusted for other factors. This suggests that women were either not informed of a delivery by SBAs due to the absence of awareness programs or if exposed, women were reluctant to adopt it. We were unable to investigate whether women were exposed to an awareness program through mass media due to unavailable data.

One limitation of our study is that we were unable to examine certain factors that were revealed in other studies. Several studies found the following associations with deliveries attended by SBAs: complications related to delivery distance to the nearest hospital, transportation issues to reach health facilities, presence of SBAs in the geographic area, and cost of SBAs [10–12, 16, 26–30]. These factors were also validated by studies in other countries [31–33]. We were unable to examine these factors due to unavailable data. Another limitation of this study is that we only analyzed the data of women who survived, and we were unable to investigate the determinants of the most affected group. The data were collected retrospectively, and the data were cross-sectional. Causality cannot be established fully as the condition may have changed after childbirth.

The main strength of this study is that the analysis is generalizable for Bangladesh as there was a large sample size accounting for the population across the entire country. To our knowledge, no other studies in Bangladesh were able to identify the divisions with fewer deliveries attended by SBAs. The response rate of the survey was high (approximately 99%), and there was little missing data. Furthermore, the possibility of a recall bias was minimized by interviewing only women with a history of childbirth within the last five years.

## Conclusions

The analysis of the 2014 BDHS data revealed that individual, fertility, and contextual variables had a significant effect on the number of deliveries attended by SBAs. A delivery by skilled personnel is required for all women in Bangladesh regardless of age, location or socioeconomic condition. From a program planning perspective, to achieve the maternal mortality target of the United Nations’ Sustainable Development Goals (SDGs) and to achieve the target of 50% of deliveries attended by SBAs of the Government of Bangladesh, it is important to consider the modifiable factors that affect the presence of SBAs during childbirth. To increase the number of deliveries attended by SBAs at the population level, community-based programs should focus on the positive factors such as ANC visits by a skilled provider and surveillance for complications during pregnancy; based on priority, health programs should include these components to increase awareness in rural areas and the divisions with a lower presence of SBAs during childbirth. Effective collaboration with stakeholders should be ensured, and further studies should be conducted to reveal unexamined factors associated with the presence of SBAs during childbirth.

## Abbreviations

ANC: Antenatal care
AOR: Adjusted odds ratio (AOR)
BBS: Bangladesh Bureau of Statistics
BDHS: Bangladesh Demographic and Health Survey
CI: Confidence interval
COR: Crude odds ratio (COR)
EA: Enumeration areas
HPNSDP: Health, population and nutrition sector development program
MDGs: Millennium development goals
MMR: Maternal mortality ratio
MOHFW: Ministry of Health and Family Welfare
OR: Odds ratio
SBA: Skilled birth attendant
SDGs: Sustainable development goals
WHO: World Health Organization
